# Characterization of chronic lung allograft dysfunction phenotypes using spectral and intrabreath oscillometry

**DOI:** 10.3389/fphys.2022.980942

**Published:** 2022-10-06

**Authors:** Anne Fu, Anastasiia Vasileva, Nour Hanafi, Natalia Belousova, Joyce Wu, Sarada Sriya Rajyam, Clodagh M. Ryan, Zoltán Hantos, Chung-Wai Chow

**Affiliations:** ^1^ Division of Respirology, Department of Medicine, Temerty Faculty of Medicine, University of Toronto, Toronto, ON, Canada; ^2^ Toronto Lung Transplant Program, Ajmera Multi-Organ Transplant Unit, University Health Network, Toronto, ON, Canada; ^3^ Toronto General Pulmonary Function Laboratory, University Health Network, Toronto, ON, Canada; ^4^ Department of Anesthesiology and Intensive Therapy, Semmelweis University, Budapest, Hungary

**Keywords:** oscillometry, lung transplant, CLAD, pulmonary function test (PFT), graft dysfunction

## Abstract

**Background:** Chronic lung allograft dysfunction (CLAD) is the major cause of death beyond 2 years after lung transplantation and develops in 50% of all patients by 5 years post-transplant. CLAD is diagnosed on the basis of a sustained drop of 20% for at least 3 months in the forced expiratory volume (FEV_1_), compared to the best baseline value achieved post-transplant. CLAD presents as two main phenotypes: bronchiolitis obliterans syndrome (BOS) is more common and has better prognosis than restrictive allograft syndrome (RAS). Respiratory oscillometry is a different modality of lung function testing that is highly sensitive to lung mechanics. The current study investigated whether spectral and intrabreath oscillometry can differentiate between CLAD-free, BOS- and RAS-CLAD at CLAD onset, i.e., at the time of the initial 20% drop in the FEV_1_.

**Methods:** A retrospective, cross-sectional analysis of 263 double lung transplant recipients who underwent paired testing with oscillometry and spirometry at the Toronto General Pulmonary Function Laboratory from 2017 to 2022 was conducted. All pulmonary function testing and CLAD diagnostics were performed following international guidelines. Statistical analysis was conducted using multiple comparisons.

**Findings:** The RAS (*n* = 6) spectral oscillometry pattern differs from CLAD-free (*n* = 225) by right-ward shift of reactance curve similar to idiopathic pulmonary fibrosis whereas BOS (*n* = 32) has a pattern similar to obstructive lung disease. Significant differences were found in most spectral and intrabreath parameters between BOS, RAS, and time-matched CLAD-free patients. *Post-hoc* analysis revealed these differences were primarily driven by BOS instead of RAS. While no differences were found between CLAD-free and RAS patients with regards to spectral oscillometry, the intrabreath metric of reactance at end-inspiration (XeI) was significantly different (*p* < 0.05). BOS and RAS were differentiated by spectral oscillometry measure R5, and intrabreath resistance at end expiration, ReE (*p* < 0.05 for both).

**Conclusion:** Both spectral and intrabreath oscillometry can differentiate BOS-CLAD from CLAD-free states while intrabreath oscillometry, specifically XeI, can uniquely distinguish RAS-CLAD from CLAD-free. Spectral and intrabreath oscillometry offer complementary information regarding lung mechanics in CLAD patients to help distinguish the two phenotypes and could prove useful in prognostication.

## Introduction

Survival following lung transplant has steadily improved. However, chronic lung allograft dysfunction (CLAD) remains the main barrier to long-term survival (Parulekar and Kao, 2019; Thabut and Mal, 2017). CLAD is defined by an irreversible decline in forced expiratory volume in 1 second (FEV_1_) to below 80% of the best baseline value achieved post-transplant and sustained for at least 3 months after other reversible causes have been ruled out (Glanville et al., 2019; Sato et al., 2011). CLAD develops in 50% of patients by 5 years after lung transplant, and once established, is associated with a poor prognosis.

CLAD manifests as two main phenotypes, bronchiolitis obliterans syndrome (BOS) and restrictive allograft syndrome (RAS) (Kotecha et al., 2020; Verleden et al., 2014; Verleden et al., 2015; Verleden et al., 2019). BOS presents with an obstructive pattern on pulmonary function testing and histopathological lesions of obliterative bronchiolitis in the small airways but no concurrent interstitial fibrosis (Meyer et al., 2014; Parulekar and Kao, 2019) whereas RAS has a restrictive pattern with peripheral lung fibrosis on histology and pleuroparenchymal opacities on computed tomography (CT) imaging (Glanville et al., 2019; Sato et al., 2011; Verleden et al., 2014). While it is less common, RAS has a worse prognosis with a survival of 6–18 months (Sato et al., 2011) compared to 3–5 years in BOS (Kulkarni et al., 2019; Parulekar and Kao, 2019). Rarely, patients can present with a mixed phenotype. Patients with BOS can also evolve to RAS, with such transitions portending worse survival (Parulekar and Kao, 2019).

Currently, there is no specific treatment for CLAD. Clinical trials of potential therapies are hampered by the fact that the diagnosis of CLAD can only be confirmed 3 months after onset (i.e., the first 20% drop in FEV_1_). Thus, the benefit of early treatment cannot be assessed. Furthermore, CLAD is a progressive disease where the decline in allograft function begins before the 80% FEV_1_ threshold is reached. While spirometry provides useful information on the CLAD development, it is insensitive and non-specific to changes in the smaller airways, where the initial pathophysiological processes of CLAD manifest (Kouri et al., 2021; Stockley et al., 2017). Studies have shown that FEV_1_ remains unchanged until 75% of the small airways are obliterated (Burgel et al., 2013; Cosio et al., 1978), by which time CLAD is well advanced. If the diagnosis of CLAD could be confirmed early using sensitive markers that can predict progression to CLAD, there is potential for earlier intervention (i.e. before or at the time of the 20% drop in FEV_1_) and the possibility of preventing the establishment of CLAD.

Oscillometry is an increasingly employed pulmonary function test (PFT) modality that is highly sensitive to changes in respiratory mechanics (Bates et al., 2011). It measures the total respiratory impedance (Zrs) during normal quiet (or tidal) breathing, expressed as the respiratory resistance (Rrs) and reactance (Xrs). Rrs reflects the viscous losses in the respiratory system (with the resistance of the airways as the main determinant) while Xrs, at the lower frequencies, reflects the elasticity of the lungs and the chest wall. Standard (also known as spectral) oscillometry provides the mean values of Zrs at different frequencies over entire breath cycles. Intrabreath oscillometry is a novel modality that tracks changes in lung mechanics continuously during inspiration and expiration, and focuses on the zero-flow instants of breathing, such as end expiration and end inspiration, where the upper airway nonlinearities are minimal (Gray et al., 2019; Chiabai et al., 2021; Hantos, 2021; Makan et al., 2022). Spectral oscillometry has been found to be more sensitive than spirometry in detecting chronic obstructive pulmonary disease (COPD) and asthma in the small airways, and is able to distinguish COPD from interstitial lung disease (Dellacà et al., 2004; Fujii et al., 2015; Paredi et al., 2010; Sugiyama et al., 2013). Our group showed that spectral oscillometry follows changes associated with biopsy-proven acute lung allograft rejection when spirometry could not (Cho et al., 2020) and intrabreath oscillometry, specifically the reactance at end inspiration (XeI) is highly correlated with two independent markers of disease severity in idiopathic pulmonary fibrosis (Wu et al., 2022).

We hypothesized that oscillometry can provide additional information at time of CLAD onset (i.e., at initial time of 20% drop in the FEV_1_) to help in the early identification of CLAD. The current study investigated whether spectral and intrabreath oscillometry can differentiate between CLAD-free, BOS, or RAS at the time of CLAD onset.

## Methods

The study was approved by the University Health Network (UHN) Research Ethics Board (REB# 17-5652). All double lung transplant recipients were eligible for enrollment. Single lung recipients, those who remained hospitalized at 3 months post-transplant, and patients who died before enrollment were excluded. Written informed consent were obtained prior to oscillometry. Oscillometry was conducted according to European Respiratory Society guidelines (King et al., 2020; Wu et al., 2020; Chang et al., 2022) using the tremoflo C-100 device (Thorasys, Montreal, QC, Canada). Spectral oscillometry was measured with the standard 5–37 Hz multifrequency signal, and intrabreath oscillometry was measured at 10 Hz frequency as previously described (Wu et al., 2022; Chang et al., 2022). A minimum of three recordings with a coefficient of variation of Rrs at 5 Hz of ≤10% was needed to pass quality control. Oscillometry was completed prior to spirometry, which were conducted without/with plethysmography as part of routine care. All testing occurred at the Toronto General Hospital (TGH) Pulmonary Function Laboratory. Lung transplant patients are followed at the TGH Pulmonary Function Laboratory weekly 3 months, then at 6, 9, 12, 18 and 24 months post-lung transplant and annually thereafter. Oscillometry is also performed prior to any additional spirometry that is requested for clinical indications.

CLAD was diagnosed and phenotyped according to International Society for Heart and Lung Transplantation guidelines (Glanville et al., 2019). Phenotyping was conducted by an experienced lung transplant physician with review of the patient’s electronic medical records, including pulmonary function tests, lung CT imaging, bronchoscopy findings and when available, histology of the lung obtained at time of retransplant or autopsy. Demographic data and clinical parameters known to affect lung function and graft rejection, including primary lung disease, donor–recipient human leukocyte antigen (HLA) match status, and cytomegalovirus (CMV) donor–recipient status, were prospectively collected and maintained in the Toronto Lung Transplant Database.

Statistical analyses were performed using Prism 6.0 (GraphPad Software) and RStudio version 4.1.1 (The R Foundation). Comparisons among groups were performed using one-way ANOVA for normal distributed variables and Kruskal–Wallis one-way analysis of variance for non-normal variables. Pearson’s chi-squared test was used for categorical variables. The data are shown as mean ± standard deviation (SD) or median and interquartile range (IQR), as appropriate. Pairwise comparisons between non-normally distributed variables were analyzed using unpaired Wilcoxon signed rank test with Bonferroni corrections.

## Results

### Demographics

**FIGURE 1:**
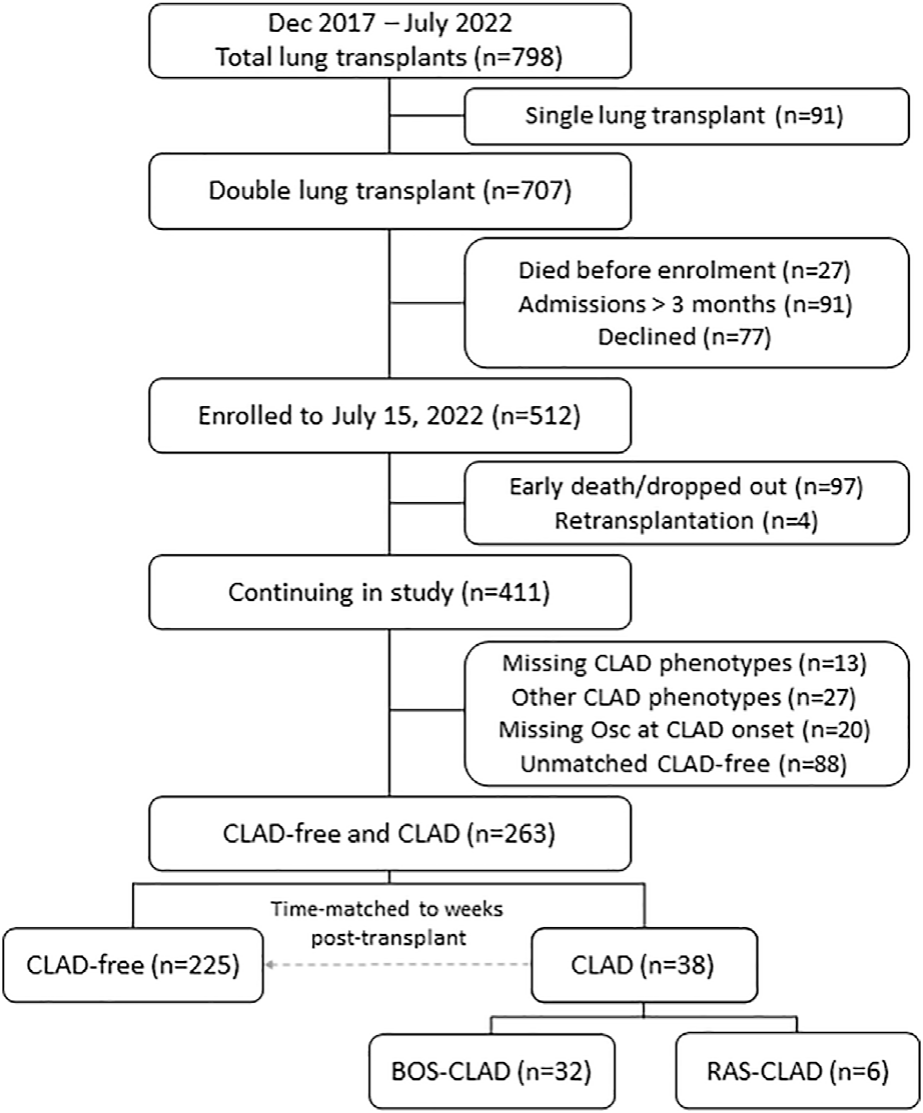
Patient recruitment, enrollment, and study cohort. Patient enrollment from December 2017 to July 2022 is shown. CLAD, chronic lung allograft dysfunction; Osc, oscillometry; BOS, bronchiolitis obliterans syndrome; RAS, restrictive allograft syndrome.

Between December 2017 to July 2022, 798 patients underwent lung transplantation. Single lung transplants (*n* = 91) were excluded (Figure 1). Of the 707 double lung transplant recipients, we excluded patients due to early death post-transplant (*n* = 27), ongoing hospitalization at 3 months (*n* = 91) or declined participation (*n* = 77). Of the 512 patients enrolled, 101 patients were excluded from our analysis due to early dropout/early death (*n* = 97) or re-transplantation (*n* = 4). CLAD developed in 85 of the remaining 411 patients; 47 participants were excluded from the current analysis due to lack of oscillometry data within 4 weeks of CLAD onset (*n* = 20), other CLAD phenotypes (*n* = 27), or insufficient data to complete CLAD phenotyping (*n* = 13). Of the 38 CLAD patients with complete data for analysis, 32 had BOS and six had RAS. Each of these 38 CLAD patients were time-matched to at least two CLAD-free lung transplant patients who had oscillometry completed within 2 weeks of the CLAD-onset dates to create a CLAD-free control group (*n* = 225).

Comparison of the CLAD-free, BOS, and RAS groups revealed their baseline clinical characteristics were similar (Table 1). The primary indications for transplant were pulmonary fibrosis, COPD and cystic fibrosis. The CLAD-free and CLAD groups were similar with respect to sex, age and height at transplant except for the body mass index which was significantly different at time of CLAD-onset or time-matched date (CLAD-free). The three groups were also similar with respect to CMV match status and immunologic risk at time of transplant (Table 1). CLAD patients were time-matched to CLAD-free patients who had an oscillometry measurement within 2 weeks of CLAD onset to control for duration of follow-up post-transplant. This duration of follow-up post-transplant was similar among CLAD-free, BOS, and RAS groups (Table 1).

**TABLE 1:** Demographics and standard pulmonary function of CLAD subjects compared to time matched CLAD-free cohort.

	CLAD-free (*n* = 225)	CLAD (*n* = 38)		*p-value*
		BOS (*n* = 32)	RAS (*n* = 6)	
Male, *n* (%)	136 (60.4)	17 (53.1)	4 (66.7)	0.688
Age, years	60.0 [49.0, 66.0]	54.5 [40.5, 61.5]	63.5 [56.8, 68.8]	0.094
Height, m	1.70 ± 0.1	1.67 ± 0.1)	1.75 ± 0.1	0.107
Weight, kg	74.5 ± 16.7	81.0 ± 16.5	87.0 ± 14.1	0.030
BMI, kg/m^2^	26.4 ± 4.9	29.0 ± 6.0	28.3 ± 4.6	0.019
PRA Positive, *n* (%)	108 (48.0)	21 (65.6)	3 (50.0)	0.175
VCM Positive, *n* (%)	56 (24.9)	9 (28.1)	0 (0.0)	0.337
ACM Positive, *n* (%)	22 (9.8)	6 (18.8)	0 (0.0)	0.212
CMV match status, *n* (%)				0.685
R^−^/D^+^ (mismatch)	102 (45.3)	18 (56.2)	2 (33.3)	
R^−^/D^−^ (negative)	45 (20.0)	6 (18.8)	2 (33.3)	
R^+^/D^+^ (positive)	78 (34.7)	8 (25.0)	2 (33.3)	
Primary disease, *n* (%)				
Pulmonary Fibrosis	85 (37.8)	10 (31.1)	1 (16.7)	0.165
COPD/Emphysema	69 (30.7)	7 (21.9)	3 (50.0)	
Cystic Fibrosis/Bronchiectasis	24 (10.7)	6 (18.8)	1 (16.7)	
Other	47 (20.9)	9 (28.1)	1 (16.7)	
Follow-up, days	265.0 [168.0, 438.0]	346.0 [195.8, 545.0]	178.5 [163.3, 319.0]	0.186
Values at CLAD-onset or time-matched date				
FVC (L)	3.04 ± 0.92	2.73 ± 1.16	2.58 ± 0.97	0.138
%predicted	75.47 ± 18.77	65.62 ± 21.44	56.77 ± 14.70	**0.002**
FEV_1_ (L)	2.34 ± 0.78	1.57 ± 0.67	2.04 ± 0.75	**<0.001**
%predicted	74.21 ± 22.30	48.28 ± 20.11	57.73 ± 11.80	**<0.001**
FEV_1_/FVC (%)	77.18 ± 12.89	58.23 ± 11.43	79.78 ± 6.31	**<0.001**
%predicted	98.16 ± 16.40	72.88 ± 14.20	103.13 ± 9.09	**<0.001**
TLC (L)	4.80 ± 1.17	4.78 ± 1.52	4.46 ± 1.03	0.825
%predicted	78.63 ± 16.47	77.09 ± 16.79	63.74 ± 15.13	0.129
RV (L)	1.72 ± 0.55	1.90 ± 0.49	1.65 ± 0.33	0.242
%predicted	88.32 ± 33.63	104.51 ± 37.28	75.46 ± 22.64	**0.039**
RV/TLC (%)	36.25 ± 8.71	41.24 ± 9.01	37.72 ± 8.36	**0.018**
%predicted	99.98 ± 29.71	122.81 ± 39.01	103.38 ± 17.22	**0.001**
DLCO (ml/min/mmHg)	15.35 ± 4.35	17.60 ± 5.50	11.17 ± 3.30	0.071
%predicted	71.57 ± 13.73	73.61 ± 16.58	65.57 ± 17.81	0.679

ACM, actual cross match; BMI, body mass index; CMV, cytomegalovirus; COPD, chronic obstructive lung disease; D, donor; DLCO, diffusing capacity for carbon monoxide; FEV_1_, forced expiratory volume in one second; FVC, forced vital capacity; PRA, panel of reactive antibodies; R, recipient; RV, residual volume; TLC, total lung capacity; VCM, virtual cross match. Continuous normal variables are reported as mean (SD); non-normal variables as median [IQR].

### Spectral oscillometry of the CLAD phenotypes

**FIGURE 2:**
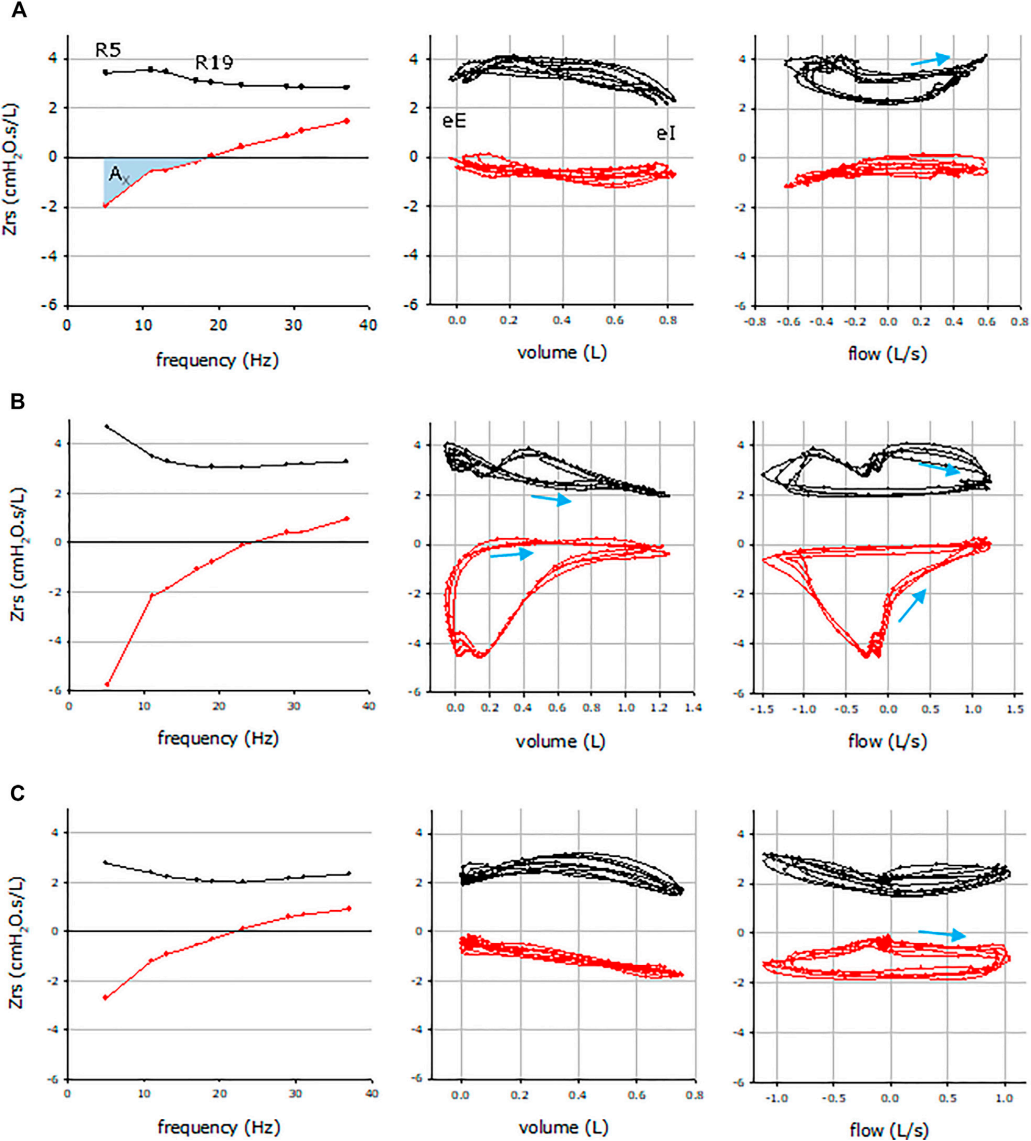
Representative respiratory impedance (Zrs) plots from a CLAD-free **(A)**, BOS **(B)** and RAS **(C)** subjects. Mean Zrs data vs. frequency (left), intrabreath Zrs data vs. volume (middle) and flow (right). Resistance and reactance data are plotted in black and red, respectively. Arrows indicate the inspiratory directions in the intrabreath loops, wherever the looping is significant. R5 and R19, resistance at 5 and 19 Hz, respectively; eE, end expiration; eI, end inspiration; AX, reactance area between 5 Hz and resonance frequency.

Figure 2 illustrates the impedance vs. frequency graphs of a CLAD-free, a BOS and a RAS patient (left panels). The pattern of the spectral oscillogram in BOS was characteristic of obstructive lung disease (Eddy et al., 2019), manifested in enhanced frequency dependence of Rrs (R5-R19) as an index of peripheral inhomogeneity with low values of X5 (reactance at 5 Hz) and high values of Ax (area of reactance), which are measures of lung elastance and ventilatory inhomogeneity, while RAS presents with a restrictive pattern that resembles idiopathic pulmonary fibrosis (Wu et al., 2022) with a primarily rightward shift in the reactance curve and moderately high Ax but normal resistance values.

Comparison of the spectral oscillometry parameters revealed significant differences in most parameters between CLAD phenotypes at CLAD-onset and those of time-matched CLAD-free patients, as shown in Table 2. *Post-hoc* analysis between BOS vs. CLAD-free and RAS vs. CLAD-free revealed that these differences were primarily observed in the 32 BOS patients (Bonferroni adjusted *p* < 0.0001 for all, Table 3) as the spectral oscillometry parameters in the six RAS patients revealed that they were similar to the CLAD-free group (Table 3). However, comparisons between BOS and RAS groups revealed significant differences in R5 at time of CLAD onset (Bonferroni adjusted *p* = 0.0234, Table 3).

**TABLE 2:** Comparison of spectral and intrabreath oscillometry parameters between CLAD-free, BOS, and RAS.

	CLAD-free (*n* = 225)	BOS (*n* = 32)	RAS (*n* = 6)	*p-value*
Spectral oscillometry				
R5 ^*a*^	3.46 [2.88, 4.40]	4.60 [3.77, 5.46]	3.55 [3.40, 3.96]	**0.0002**
R5-19 ^*a*^	0.57 [0.23, 1.05]	1.30 [0.88, 1.77]	0.89 [0.75, 1.37]	**<0.0001**
X5 ^*a*^	−1.58 [−2.21, −1.18]	−2.91 [−4.81, −2.15]	−2.41 [−2.87, −1.55]	**<0.0001**
Ax ^*b*^	9.0 [5.0, 17.1]	22.6 [13.8, 38.5]	18.1 [10.4, 18.8]	**<0.0001**
Fres (Hz)	18.3 [13.8, 22.0]	24.1 [21.0, 28.2]	21.6 [18.1, 23.4]	**<0.0001**
Intrabreath Oscillometry				
ReE ^*a*^	2.84 [2.32, 3.67]	3.92 [3.25, 4.39]	2.65 [2.37, 3.18]	**0.0004**
ReI ^*a*^	2.29 [1.91, 2.80]	2.58 [2.21, 3.22]	2.25 [1.95, 2.52]	**0.0258**
XeE ^*a*^	−0.24 [−0.67, 0.08]	−1.44 [−2.44, −0.61]	−0.62 [−1.78, −0.35]	**<0.0001**
XeI ^*a*^	−0.30 [−0.61, −0.10]	−0.74 [−1.27, −0.48]	−0.84 [−1.28, −0.62]	**<0.0001**
ARV ^*c*^	−0.22 [−0.50, 0.01]	−0.24 [−0.53, 0.08]	−0.27 [−0.37, −0.13]	0.8967
ARV′ ^*d*^	1.44 [0.93, 2.38]	2.62 [1.90, 3.32]	1.76 [1.15, 2.20]	**<0.0001**
AXV ^*c*^	0.30 [0.12, 0.74]	0.69 [0.34, 1.40]	0.42 [0.29, 0.59]	**0.0006**
AXV′ ^*d*^	−0.07 [−0.53, 0.20]	−1.19 [−2.52, −0.50]	−0.08 [−2.33, 0.30]	**<0.0001**

Units of measures: ^*a*^ cmH_2_O∙s/L; ^*b*^ cmH_2_O/L; ^*c*^ cmH_2_O∙s; ^*d*^ cmH_2_O.

R5, resistance at 5 Hz; R19, resistance at 19 Hz; R5–19, difference in resistance between 5 and 19 Hz; Ax, reactance area between 5 Hz and Fres; Fres, resonance frequency; ReE, resistance at end-expiration; ReI, resistance at end-inspiration; XeE, reactance at end-expiration; XeI, reactance at end-inspiration; ARV, area of resistance volume loop; ARV’, area of resistance flow loop; AXV, area of reactance volume loop; AXV’, area of reactance flow loop. Data are shown as median [IQR]. Statistics were performed with Kruskal–Wallis one-way analysis of variance.

**TABLE 3:** Table of *p*-values from paired comparisons with Wilcoxon signed rank test with Bonferroni correction for multiple comparisons.

			BOS vs. CLAD-free		RAS vs. CLAD-free		BOS vs. RAS	
Spectral oscillometry								
R5			**<0.0001**		0.7925		**0.0234**	
R5-19			**<0.0001**		0.1665		0.2285	
X5			**<0.0001**		0.2458		0.2133	
Ax			**<0.0001**		0.2262		0.1987	
Fres			**<0.0001**		0.1823		0.2446	
Intrabreath Oscillometry								
ReE			**0.0001**		0.7877		**0.0410**	
ReI			**0.0092**		0.6183		0.0816	
XeE			**<0.0001**		0.1255		0.2970	
XeI			**<0.0001**		**0.0170**		0.7108	
ARV			0.6694		0.8357		1.0000	
ARV′			**<0.0001**		0.4230		0.1591	
AXV			**0.0001**		0.5058		0.2285	
AXV′			**<0.0001**		0.9728		0.1987	

For definitions and units, *see* legend to Table 2.

### Intrabreath oscillometry and CLAD

Intrabreath oscillometry at the time of CLAD onset (or the time-matched date in the CLAD-free group) was also significantly different amongst CLAD-free, BOS, and RAS patients (Table 2). These were observed in the post-hoc analysis of the resistance measurements at end-inspiration (ReI) and end-expiration (ReE) as well as the reactance values at end-inspiration (XeI) and end-expiration (XeE) (Table 3). Similarly, nearly all intrabreath oscillometry parameters, with the exception of the area of resistance-volume loop (ARV), were significantly different between BOS and its time-matched CLAD-free cohort (Table 3). In contrast to the findings of spectral oscillometry, the intrabreath metric of reactance at end-inspiration, XeI, was significantly different between the RAS-CLAD and the CLAD-free group (Bonferroni adjusted *p* = 0.0170, Table 3). Comparisons between BOS and RAS groups revealed significant differences in ReE at time of CLAD onset (Bonferroni adjusted *p* = 0.0410, Table 3).

The intrabreath impedance-volume loops (Figure 2, middle panels) and impedance-flow loops (Figure 2, right panels) are markedly different in the patients who remained CLAD-free (A) and those who had BOS (B) or RAS (C). In CLAD-free patients (A), there is minimal change in the resistance and reactance values during normal tidal inspiration and expiration. In contrast, patients with BOS (B) exhibit progressively lower resistance values during inspiration that recovers during expiration while there is a markedly steep drop in the reactance values as the breath reaches end expiration. This club shaped reactance-volume loop is characteristic of expiratory flow limitation observed in obstructive lung disease. In patients with RAS (C, middle panel), the main finding is the thin reactance-volume loop and linear decrease in the reactance values during inspiration that is followed with the same slope of increase during expiration, while the resistance-flow loop is similar to that observed in the CLAD-free state (A, middle panel).

The differences in the resistance and reactance measurements at end-inspiration and end-expiration, at a state of zero flow, is illustrated in the impedance-flow loops (Figure 2, far right panels). The reactance-flow loops are markedly different amongst the CLAD-free (A), BOS (B) and RAS (C) states, where the strong negative volume dependence of reactance resulted in low end-inspiratory reactance (a restrictive feature) and clockwise looping in the reactance-flow diagram in RAS as opposed to the counter-clockwise pattern in BOS. In contrast, the differences in the resistance-flow loops were less evident.

## Discussion

This is the first study to characterize lung transplant patients at the time of CLAD onset by both standard (spectral) and intrabreath oscillometry. We observed distinct spectral oscillogram patterns among CLAD-free, BOS and RAS as well as significant differences in both spectral and intrabreath oscillometry parameters that can distinguish CLAD and its phenotypes from CLAD-free states at the time of CLAD onset. By matching the date of the oscillometry/pulmonary function test of the CLAD-free patients to within 2 weeks of the CLAD-onset date for the CLAD patients, we were able to account for the time-dependence of changing lung function post-transplant, allowing us to compare CLAD and CLAD-free patients with similar periods of follow-up time post-lung transplant.

We observed significant differences in both spectral and intrabreath oscillometry parameters depending on CLAD status. Larger differences were found in the BOS than the RAS group compared to CLAD-free. BOS and RAS were different with respect to the spectral oscillometry parameter, R5, and the intrabreath oscillometry metric, ReE. BOS is an obstructive disease that develops from progressive obliteration of the small airways and presents with a concurrent drop in the FEV_1_/FVC (forced vital capacity) ratio at time of CLAD-onset (Glanville et al., 2019; Meyer et al., 2014) (Table 1). This is reflected in the pattern of the resistance and reactance curves that resemble those of patients with COPD, with high R5-19 and Ax, and low X5 values (Eddy et al., 2019; Hantos, 2021). The intrabreath oscillometry in BOS-patients reveals dynamic changes in airway closure and gas-trapping during tidal breathing with significantly higher ARV’ (area of resistance-flow volume loop) and AXV’ (area of reactance-flow loop), respectively. In contrast, RAS develops as a consequence of fibroelastosis of the lung allograft (Glanville et al., 2019; Verleden et al., 2019) and presents with restrictive physiology (Table 1) and an oscillometry pattern that is similar to patients with pulmonary fibrosis (Sugiyama et al., 2013; Wu et al., 2022) that include normal resistance measurements and a right-ward shift in the reactance curve, with increased Ax and low X5. The intrabreath oscillometry measurements in RAS are notable for the markedly abnormal XeI, that reflects increased distension at end-inspiration in the context of a stiff fibrotic lung. In the pulmonary fibrosis study, XeI was found to be the single oscillometry parameter most correlated with FVC and total lung capacity (TLC) (Wu et al., 2022).

The demographic and clinical characteristics of the patients at time of transplant were similar for the CLAD-free, BOS and RAS patients. The most common manifestation of CLAD is BOS and only up to 30% of CLAD patients develop RAS (Sato et al., 2011). Our study cohort is similar to those previously reported (Sato et al., 2011), with 20% of our patients being classified with the RAS phenotype. The median follow-up time in the current study is less than 1 year.

The short follow-up is a major study limitation and the main reason for the relatively small number of CLAD cases, particularly those with RAS. Since CLAD occurs in a time-dependent manner, the number of patients with RAS and/or BOS will increase with longer follow-up and ongoing recruitment of patients. A larger RAS patient cohort will improve the generalizability of the results and the power of the study. With longer follow-up and larger cohort, the analysis of the oscillometry data may show differences that are currently undetected between RAS and CLAD-free patients.

The current methods for diagnosis of CLAD are not without limitations. Despite the published consensus guidelines used to define CLAD, the threshold drop in spirometry (currently defined as persistent ≥20% decline of at least 12 weeks in FEV_1_ compared to the baseline value achieved post-transplant) is subject to controversy due to the differing frequency of pulmonary function testing in various centres, although they clearly have an impact on the diagnosis and date of CLAD-onset (Glanville et al., 2019). Moreover, the extent of investigations to exclude other causes for the drop in lung function is not defined and differs amongst transplant centers (Glanville et al., 2019). Many lung transplant centers also follow patients with spirometry only, complicating the diagnosis of RAS, as restrictive defects can only be made with the additional measurements of lung volumes. This can be further exacerbated when the BOS patients transition into the mixed phenotype, where spirometry, lung volumes and chest imaging are needed to detect this development.

The diagnosis of CLAD in the absence of pulmonary function testing is not recommended, although patients can present with imaging or pathologic findings that are characteristic for CLAD but are too sick to perform spirometry, as accurate measurements require forced expiratory maneuvers that must be repeated in order to meet technical quality control standards. Oscillometry is particularly attractive in this clinical context as it is performed during normal quiet breathing and can be tolerated by anyone who can breathe while wearing a nose clip. Furthermore, the commercial oscillometry devices are small, portable, and easily deployed in a clinic setting or at the bedside. While strict quality control and assurance standards are needed for accurate measurements of oscillometry, no special expertise or infrastructure is required. As such, implementation of oscillometry for lung function assessment post-lung transplant improves accessibility for patients who cannot visit diagnostic pulmonary function laboratories due to mobility issues or who live in remote, underserviced regions of the country.

The need for non-invasive, sensitive methods for early detection CLAD has stimulated much research. Many of these offer promise but are hampered by need for technical expertise and infrastructure support and/or are invasive. They include multi-breath nitrogen wash-out to evaluate small airway function, different imaging modalities and analytic strategies (e.g. high-resolution computed tomography), novel assessments of immune cell counts, chemokines, cytokines, and circulating cell free DNA (Belloli et al., 2021; Shtraichman and Diamond, 2020; Tissot et al., 2019; Veraar et al., 2021). Some groups are also investigating computational models to estimate a recipient’s risk of CLAD using clinical and multi-omic databases (Koutsokera et al., 2017; Pison et al., 2014).

These new techniques and tools, including oscillometry, need further validation and standardization before an individual patient approach can be developed. Timely diagnosis and accurate distinction between CLAD phenotypes are important as they would allow for earlier intervention and offers the possibility of evaluating specific therapies for the early treatment of CLAD. Early diagnosis of RAS is particularly beneficial as these patients have a lower survival rate than BOS (Kulkarni et al., 2019; Todd et al., 2014).

In conclusion, both spectral and intrabreath oscillometry can differentiate between patients with CLAD and those who are remaining CLAD-free and can clearly distinguish between different CLAD phenotypes at the time of CLAD onset. Significant changes in multiple spectral and intrabreath oscillometry parameters may indicate BOS development whereas significant changes observed only in XeI can be an early biomarker to identify RAS. The frequency of lung function monitoring decreases with longer durations post-transplant. More frequent follow-up with oscillometry, regardless of duration post-transplant and evaluation of a larger patient population are needed to demonstrate the potential of oscillometry as a sensitive measure of early diagnosis of CLAD, i.e. at the time of or before the first drop in the FEV_1_.

## Data Availability

The raw data supporting the conclusions of this article will be made available by the authors, without undue reservation.
